# Supplementary material for: Hedgehog-GLI and Notch Pathways Sustain Chemoresistance and Invasiveness in Colorectal Cancer and Their Inhibition Restores Chemotherapy Efficacy
Source: Cancers (Basel). 2023 Feb 25;15(5):1471. doi: 10.3390/cancers15051471 (PMC10000782; doi:10.3390/cancers15051471)
Supplement: Supplementary file 1 [file cancers-15-01471-s001.zip › cancers-2205254-supplementary.pdf]

Supplementary Table S1. Primers for gene expression

	FORWARD	REVERSE
MUCIN 2	CGAAACCACGGCCCACCCCGT	GACCACGGCCCCGTTAAGCA
AXIN	CTCCTTACGTGTGGGCAGT	CTTCATCCTCTCGCATCTGC
E-CADHERIN	TCTTCAATCCGACCACGTTACAA	TATTGGGGGCATCAGCATCAG

Supplementary figure S1

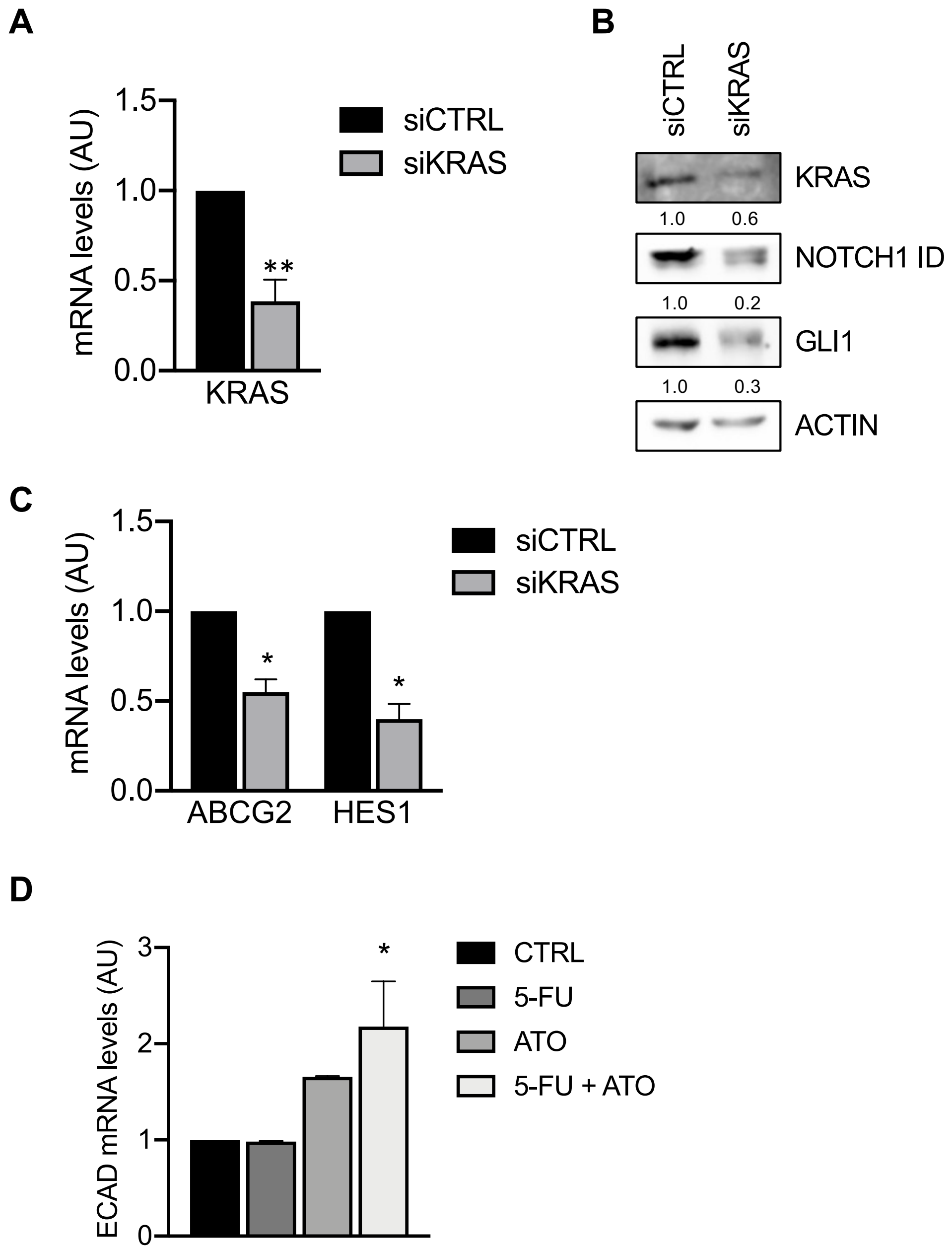

Supplementary figure S2

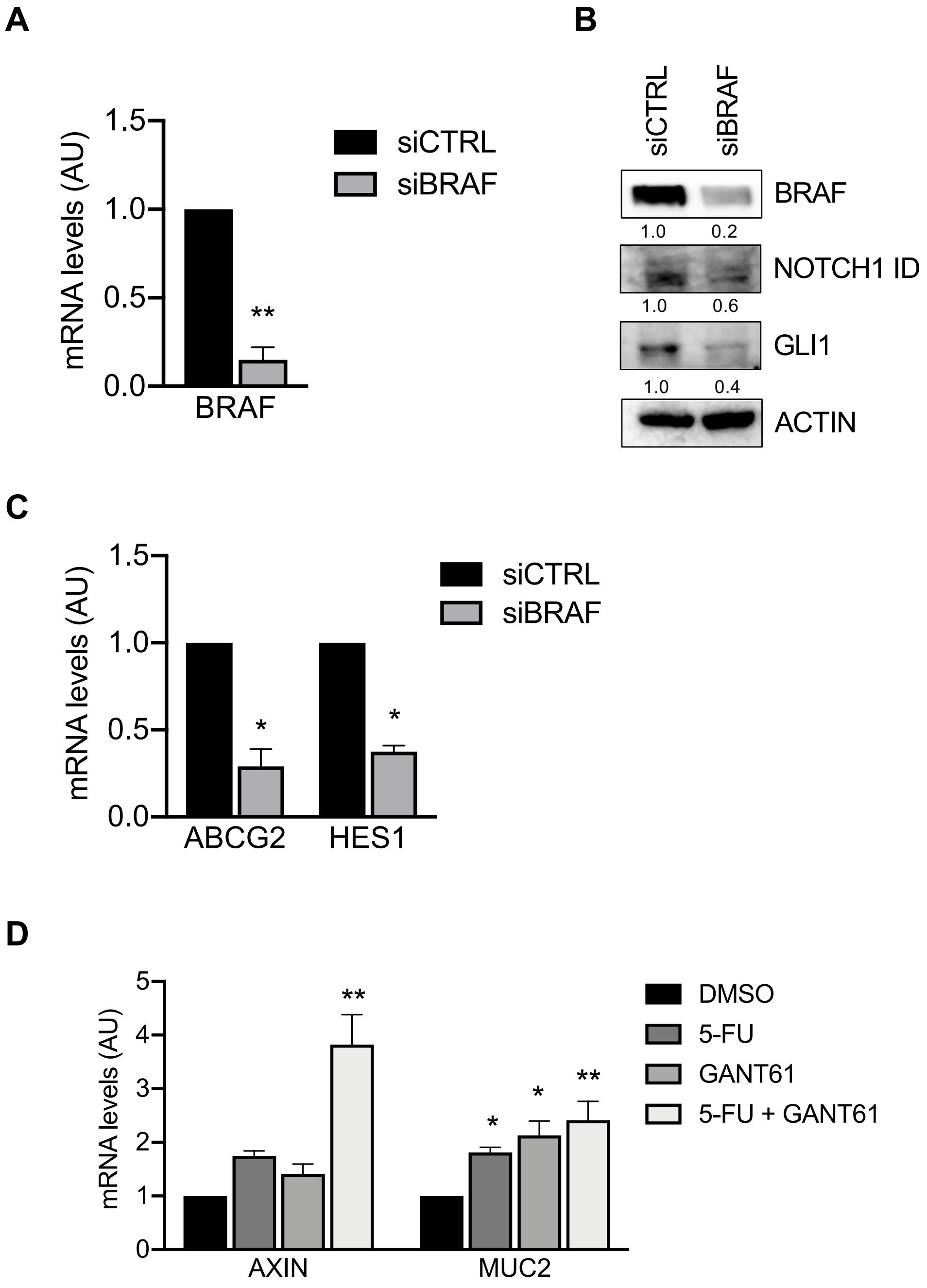

Supplementary figure S3

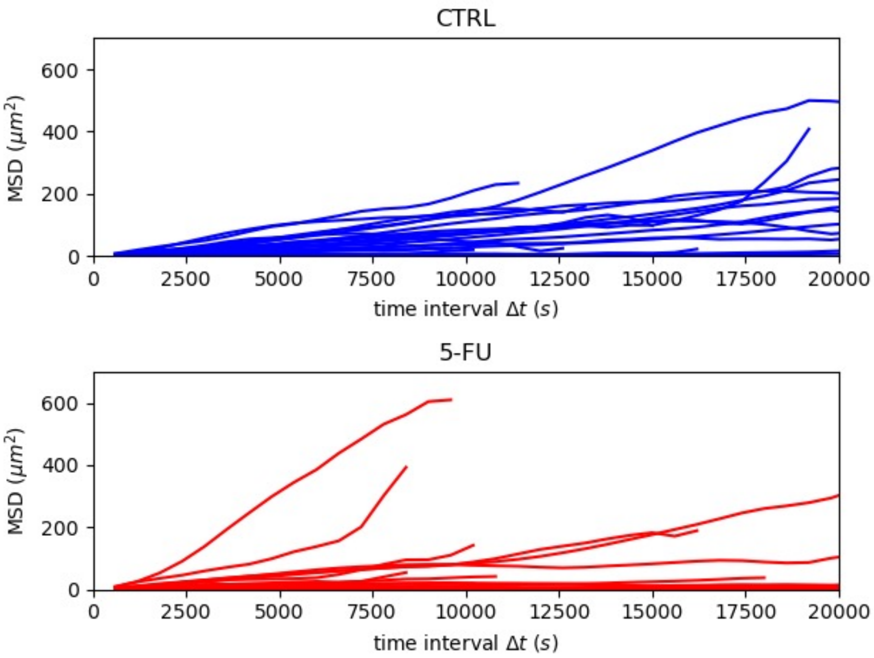

Supplementary figure S4: uncropped full scan for figure 1

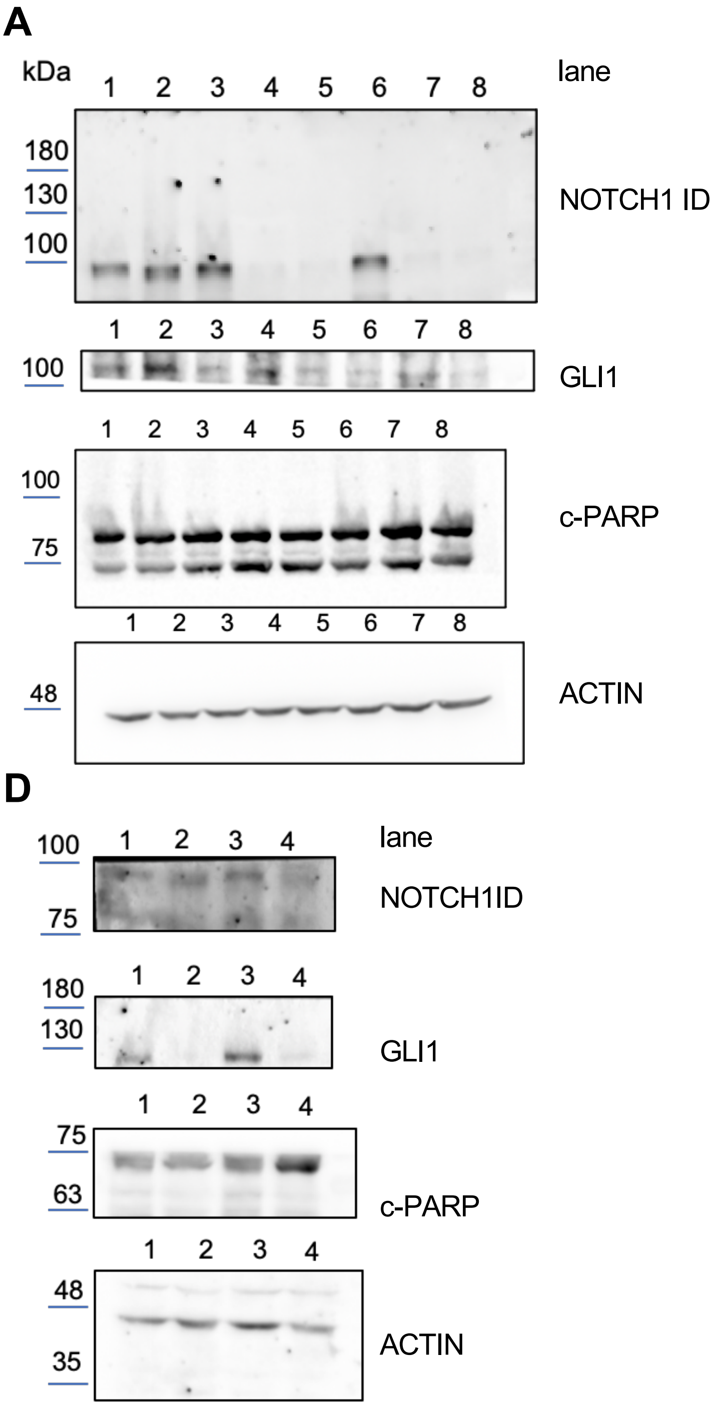

Supplementary figure S5: uncropped full scan for figure 2

A

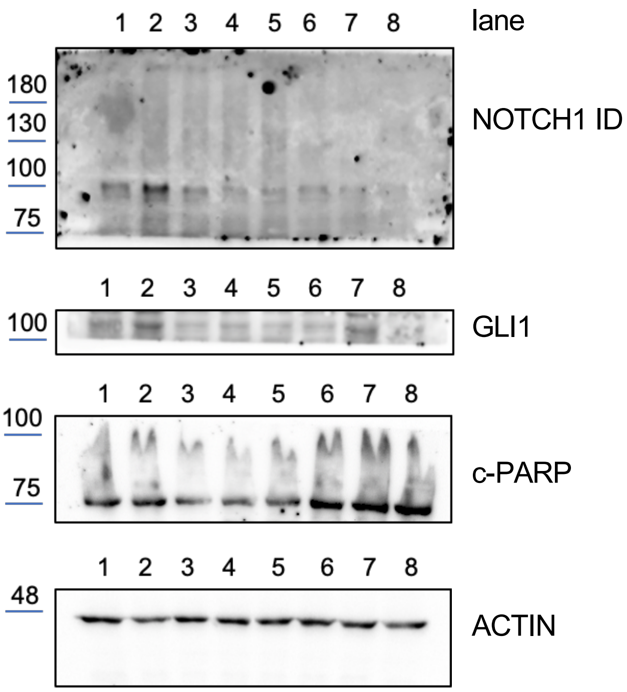

Supplementary: uncropped full scan for supplementary figures

Suppl Figure S1B

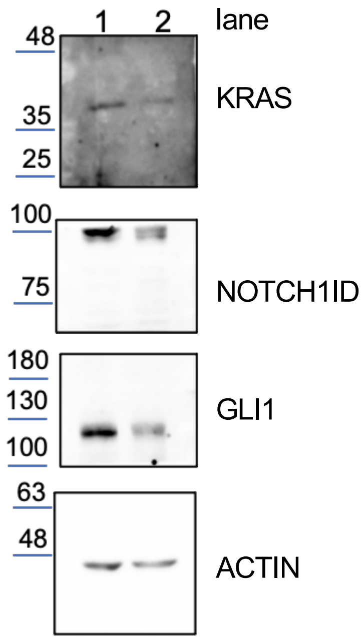

Suppl Figure S2B

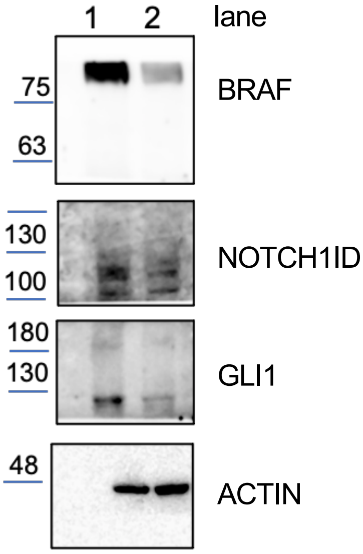

Supplementary material

Supplementary Table S1. List of gene expression primers for quantitative real-time PCR. Gene expression of GLI1, HES1, c-MET, ABCG2, CD133, KRAS, BRAF, HPRT, GAPDH and β-ACTIN was assessed using Life technologies "best coverage" assays (Life Technologies).

Supplementary Figure S1. (A) Quantitative Real Time PCR of KRAS in HCT116 cells after KRAS silencing (siKRAS) and control group (siCTRL). **(B)** Western blot analysis of KRAS, GLI1 and NOTCH1 ID in HCT116 cells after KRAS silencing (siKRAS) and control group (siCTRL). **(C)** Quantitative Real Time PCR of ABCG2 and HES1 in HCT116 cells after KRAS silencing (siKRAS) and control group (siCTRL). **(D)** mRNA levels of E-cadherin (ECAD) expressed in arbitrary units in HCT116 treated with 5-FU, ATO, combined treatment and control group (CTRL). Data are representative of three independent experiments *$p < 0.05$ versus control; **$p<0.01$ versus 5-FU (two-way ANOVA test).

Supplementary Figure S2. (A) Quantitative Real Time PCR of BRAF in HCT116 cells after BRAF silencing (siBRAF) and control group (siCTRL). **(B)** Western blot analysis of GLI1 and NOTCH1 ID in HT29 cells after BRAF silencing (siBRAF) and control group (siCTRL). **(C)** Quantitative Real Time PCR of ABCG2 and HES1 in HT29 cells after BRAF silencing (siBRAF) and control group (siCTRL). **(D)** mRNA levels of AXIN and MUC2 expressed in arbitrary units, in HT29 treated with 5-FU, GANT61, the combined treatment and control group (CTRL). Data are representative of three independent experiments *$p<0.05$; **$p<0.01$ (two-way ANOVA test)

Supplementary Figure S3. The plots show how the mean square displacements (MSD) changes for different time intervals (Δt) for each tracked single cell trajectory from either the HCT116 control group 3D organoids (CTRL) or the HCT116 5-FU treated group (5-FU).

Supplementary Figure S4. uncropped full scan for figure 1 (panels refer to main figure panel).

Supplementary Figure S5. uncropped full scan for figure 2 (panels refer to main figure panel).

Supplementary Figure S6. uncropped full scan for supplementary figures.

Supplementary videos. time lapse of gfp transduced oHCT116 at basal state (oHCT116-gfp CTRL) and treated with 5-FU (oHCT116-gfp 5-FU); length: 6 hours
